# Harnessing ROCK biology to revolutionize diabetic nephropathy: decoding mechanisms, designing therapies

**DOI:** 10.1007/s13340-025-00861-7

**Published:** 2025-11-25

**Authors:** Keiichiro Matoba

**Affiliations:** Division of Diabetes, Metabolism and Endocrinology, Department of Internal Medicine, The Jikei University School of Medicine, 3-25-8, Nishishimbashi, Minato-ku, Tokyo, 105-8461 Japan

**Keywords:** Diabetes, Chronic kidney disease, Diabetic nephropathy, Rho, Associated, Coiled, Coil, Containing protein kinase (ROCK)

## Abstract

Diabetes remains a major cause of kidney failure globally, presenting substantial challenges to healthcare systems worldwide. Although significant progress has been made in understanding its pathogenesis, residual risks persist despite current therapies. Emerging evidence underscores the pivotal role of small GTPases—particularly Rho and Rho-associated coiled-coil-containing protein kinase (ROCK)—in the progression of diabetic nephropathy. This comprehensive review consolidates current knowledge on the distinct pathophysiological roles of the ROCK isoforms, ROCK1 and ROCK2, in diabetic nephropathy, drawing on recent insights from both genetic and pharmacological studies. We explore how ROCK signaling interfaces with key pathological mechanisms, including podocyte injury, glomerulosclerosis, tubular dysfunction, and metabolic disturbances. Particular emphasis is placed on isoform-specific functions: ROCK1 primarily regulates AMP-activated protein kinase-mediated fatty acid metabolism and mitochondrial dynamics, while ROCK2 modulates peroxisome proliferator-activated receptor α signaling and inflammatory responses. Furthermore, we discuss the translational implications of these findings, focusing on the therapeutic potential of ROCK inhibitors in chronic kidney disease (CKD) with diabetes and related disorders, such as focal segmental glomerulosclerosis, as well as their impact on electrolyte balance. By integrating molecular insights with clinical considerations, this review provides a framework for developing targeted strategies to halt the CKD progression in people with diabetes.

## Introduction

People with diabetes and chronic kidney disease (CKD) are at high risk for both kidney failure and atherosclerotic cardiovascular disease. Although current standard-of-care treatments—such as renin–angiotensin system inhibitors and sodium–glucose cotransporter 2 inhibitors—provide clinical benefits, they often fail to fully halt CKD progression in individuals with diabetes [1]. This limitation underscores the urgent need for more effective therapeutic strategies. In response, research has increasingly focused on novel molecular targets, particularly small GTPases and their effectors, which are now recognized as critical regulators of diabetic nephropathy and other proteinuric kidney disorders, including focal segmental glomerulosclerosis (FSGS).

The Rho family of small GTPases—including RhoA, Rac1, and Cdc42—belongs to the Ras superfamily and acts as a group of molecular switches that alternate between active (GTP-bound) and inactive (GDP-bound) states. These proteins regulate a wide range of cellular functions, including cytoskeletal organization, cell adhesion, migration, and gene expression. Rho-associated coiled-coil-containing protein kinases (ROCK1 and ROCK2) are principal downstream effectors of Rho GTPases, mediating extracellular signals by phosphorylating various intracellular substrates.

Recent research has clarified the distinct roles of ROCK isoforms in kidney physiology and disease. Although ROCK1 and ROCK2 share high homology within their kinase domains, they differ in modes of activation and functional roles [2–4]. In diabetic kidneys, both isoforms become aberrantly activated, contributing to structural and functional deterioration through multiple mechanisms [5, 6]. Similarly, in animal models of FSGS, ROCK upregulation in podocytes drives injury and glomerular scarring, underscoring the pathway’s broader role in glomerulopathies [7]. This review offers a comprehensive analysis of ROCK signaling in diabetic nephropathy, with insights into its relevance for CKD from non-diabetic etiologies, synthesizing established knowledge with recent findings from genetic and pharmacological studies.

## Molecular structure and regulation of ROCK isoforms

**Fig. 1:**
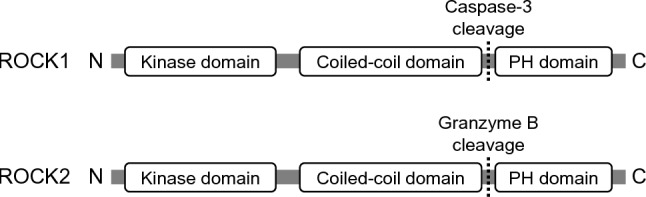
Structure of ROCK1 and ROCK2 isoforms. Both isoforms share a conserved domain architecture comprising an N-terminal kinase domain with the catalytic cleft, a central coiled-coil region containing the Rho-binding domain, and a C-terminal pleckstrin homology (PH) domain with an embedded cysteine-rich motif. Key structural differences emerge in the C-terminal regulatory region, where ROCK1 contains a caspase-3 cleavage motif while ROCK2 possesses both a granzyme B cleavage site

ROCK1 and ROCK2 are serine/threonine kinases and play pivotal roles in diverse cellular signaling pathways [8]. These evolutionarily conserved kinases possess a sophisticated multi-domain architecture that enables precise regulation of their enzymatic activity and biological functions [9]. Structurally, ROCK comprises three characteristic domains: an N-terminal catalytic domain, a central coiled-coil domain, and a C-terminal pleckstrin homology (PH) domain containing a cysteine-rich insertion (Fig. 1). Similar to other members of the myotonic dystrophy kinase subgroup, ROCK requires both N-terminal and C-terminal extension segments flanking its core catalytic domain for full enzymatic activity. The central coiled-coil domain contains the Rho-binding domain, which interacts specifically with GTP-bound forms of Rho proteins. Structural studies have demonstrated that Rho binding induces significant conformational changes in this domain, which are essential for ROCK activation. Under basal conditions, the C-terminal region folds back to interact with the N-terminal kinase domain, maintaining the protein in an autoinhibited conformation. This intramolecular interaction serves as a key regulatory mechanism to suppress aberrant kinase activity under resting conditions [10].

Despite structural similarities, ROCK1 and ROCK2 exhibit distinct regulatory features that underpin their functional divergence. ROCK1 is preferentially activated via caspase-3-mediated cleavage during apoptosis [3]. This cleavage occurs at a conserved DETD sequence within the C-terminal region and is implicated in membrane blebbing and the formation of apoptotic bodies. By contrast, ROCK2 is predominantly activated by granzyme B cleavage at an IETD site during immune responses [4]. These isoform-specific cleavage patterns suggest differential activation in response to distinct cellular stressors or pathological stimuli.

In the kidney, both ROCK isoforms are broadly expressed. Immunohistochemical analyses and single-cell RNA sequencing have shown that ROCK1 and ROCK2 are expressed in glomerular podocytes, mesangial cells, endothelial cells, tubular epithelial cells, and proximal tubules [5, 7]. ROCK1 is found in both cytoplasmic and membrane-associated compartments, which indicates its roles in cell adhesion and contractility. Notably, ROCK2 exhibits stronger nuclear localization compared to ROCK1, which may be associated with its emerging role in transcriptional regulation [11]. Indeed, nuclear ROCK2 has been shown to phosphorylate histone modifiers and transcription factors, suggesting potential involvement in epigenetic regulation. These structural and regulatory distinctions between ROCK isoforms have significant implications for kidney physiology and disease. The divergent activation mechanisms, interaction partners, and subcellular distributions enable ROCK1 and ROCK2 to mediate different aspects of kidney function, from maintaining the glomerular filtration barrier to modulating tubular transport. A detailed understanding of these molecular differences is essential for developing selective therapeutic strategies targeting ROCK-dependent pathways in kidney diseases.

## ROCK activation in diabetic nephropathy

The dysregulation of ROCK signaling has emerged as a central molecular mechanism underlying the pathogenesis of diabetic nephropathy. Studies from both experimental models and a pilot clinical study demonstrate that the diabetic microenvironment fosters persistent ROCK activation through multiple interconnected pathways, collectively contributing to progressive kidney injury [12, 13].

At the cellular level, high glucose condition is a principal driver of ROCK pathway dysregulation, activating several biochemical cascades simultaneously. Elevated glucose concentrations directly enhance ROCK activity in kidney cells, inducing the expression of fibronectin via an activator protein-1-dependent mechanism [14]. This glucose-driven ROCK activation is particularly evident in podocytes and mesangial cells, where it leads to the phosphorylation of downstream substrates such as myosin phosphatase target subunit 1 and myosin light chain [15]. Additionally, metabolic disturbances promote the accumulation of transforming growth factor-β (TGF-β), which activates the ROCK signaling, inducing kidney fibrosis in the setting of diabetes [16, 17].

Hemodynamic alterations typical of diabetic nephropathy represent another critical axis of ROCK activation [18]. Glomerular hyperfiltration and hypertension impose biomechanical stress on kidney cells, activating RhoA through mechanosensitive guanine nucleotide exchange factors. Angiotensin II, a major contributor to diabetic kidney injury, exacerbates this process via AT1 receptor-mediated stimulation of Rho/ROCK signaling, linking the renin-angiotensin system to cytoskeletal remodeling and cellular contraction [19, 20]. These hemodynamic forces are particularly implicated in early podocyte injury and glomerular barrier dysfunction, where ROCK activation disrupts the architecture of foot processes and slit diaphragms.

Lipid abnormalities associated with diabetes also act as potent activators of ROCK signaling in the kidney. Lysophosphatidic acid, a pro-inflammatory lipid mediator produced and released by activated platelets, is found at elevated levels in various pathological conditions, including diabetes and acute coronary syndrome [21, 22]. Lysophosphatidic acid initiates intracellular signaling cascades that converge on ROCK activation [23]. Similarly, bioactive sphingolipids such as sphingosine-1-phosphate directly modulate ROCK activity and have been shown to induce epithelial-mesenchymal transition in kidney tubular cells [24]. These lipid-mediated pathways not only directly activate ROCK but also act synergistically with hyperglycemia and angiotensin II signaling, thereby sustaining a vicious cycle of kidney injury.

The pathological consequences of sustained ROCK activation in diabetic nephropathy are profound and multifaceted. In podocytes, excessive ROCK activity induces mitochondrial fission, leading to foot process effacement, loss of slit diaphragm integrity, and albuminuria while concurrently promoting apoptosis through Notch-dependent pathways [15, 17]. In mesangial cells, ROCK signaling induces phenotypic transformation, stimulates extracellular matrix production, and drives proinflammatory cytokine secretion, all of which contribute to glomerulosclerosis [25, 26].

Clinical observations corroborate these experimental findings. Immunohistochemical analysis of kidney biopsies from patients with diabetes consistently reveals increased ROCK activity in both the glomeruli and the tubulointerstitium compared to individuals without diabetes [13]. These collective findings position ROCK inhibition as a disease-modifying strategy capable of targeting multiple pathological axes in diabetic nephropathy, potentially altering its natural progression.

## Isoform-specific functions of ROCK1 in diabetic nephropathy

The distinct pathological contributions of ROCK1 to diabetic nephropathy have been increasingly elucidated through research into its metabolic regulatory functions. As one of the two ROCK isoforms, ROCK1 has emerged as a key regulator of cellular energy homeostasis in kidney cells, particularly within the glomerular compartment, where it exerts profound effects on mitochondrial dynamics and lipid metabolism [5, 15].

Central to ROCK1-mediated metabolic disruption is its interference with AMP-activated protein kinase (AMPK) signaling—a critical energy-sensing pathway that maintains cellular homeostasis under stress [27]. In the diabetic kidney, persistent activation of ROCK1 suppresses AMPK activity through phosphorylation-dependent mechanisms that diminish its catalytic function. This inhibition precipitates a metabolic imbalance, simultaneously impairing energy production while increasing energy expenditure. The downstream consequences are most evident in fatty acid oxidation pathways, where ROCK1-mediated AMPK suppression downregulates key effectors such as peroxisome proliferator-activated receptor γ coactivator 1α (PGC1α), a master regulator of mitochondrial biogenesis, and carnitine palmitoyltransferase 1A (CPT1A), the rate-limiting enzyme for fatty acid transport into mitochondria.

ROCK1 also exerts detrimental effects on mitochondrial structure and function. Under hyperglycemic conditions, ROCK1 directly phosphorylates dynamin-related protein 1, enhancing its GTPase activity and promoting excessive mitochondrial fission [15]. This imbalance between mitochondrial fission and fusion undermines mitochondrial quality control and bioenergetic efficiency. The resulting fragmented mitochondria exhibit impaired oxidative phosphorylation, reduced membrane potential, and elevated production of reactive oxygen species. In podocytes—highly specialized cells with limited regenerative capacity—ROCK1-driven mitochondrial dysfunction leads to energetic failure and oxidative damage, contributing to the onset of albuminuria, a hallmark of CKD in people with diabetes. Genetic models strongly support the pathogenic role of ROCK1 in diabetic nephropathy. Podocyte-specific knockin mice expressing a constitutively active mutant of ROCK1 developed significant albuminuria, while maintaining normal metabolic parameters (blood pressure, glucose, creatinine). Histological analysis revealed mesangial matrix expansion and prominent podocyte foot process effacement, as observed by electron microscopy, accompanied by significant podocyte loss. These findings establish that podocyte-specific ROCK1 activation drives key features of diabetic nephropathy, including albuminuria, structural podocyte damage, and extracellular matrix changes, independent of systemic metabolic alterations.

Similarly, systemic ROCK1-deficient mice show improved kidney outcomes in streptozotocin-induced diabetes, with partial protection against albuminuria and tubulointerstitial fibrosis [28]. These effects were accompanied by the preserved expression of megalin and cubilin, along with attenuated upregulation of TGF-β and interleukin-1β. These genetic studies have helped differentiate the roles of ROCK1 from those of ROCK2, revealing that while both contribute to diabetic kidney injury, their mechanisms and downstream targets differ. The protective effects observed in ROCK1-deficient models appear to arise primarily from the preservation of metabolic homeostasis, rather than direct modulation of inflammation or fibrosis, underscoring ROCK1’s specialized role in kidney energy regulation.

## ROCK2 in inflammation and fibrosis

ROCK2 has emerged as a critical mediator of inflammation and fibrosis in the pathogenesis of diabetic nephropathy. At the molecular level, ROCK2 functions as a potent activator of nuclear factor kappa B (NF-κB) signaling in kidney cells, particularly mesangial cells and endothelial cells [23, 29]. Under diabetic conditions, inflammatory signaling is markedly upregulated in the kidney, promoting the phosphorylation and subsequent degradation of IκBα, the inhibitory protein that normally sequesters NF-κB in the cytoplasm. This degradation permits the nuclear translocation of NF-κB, where it drives the transcription of various proinflammatory genes, including monocyte chemoattractant protein-1 (MCP-1) and tumor necrosis factor-alpha (TNF-α). ROCK2 plays a critical role in facilitating this nuclear translocation of NF-κB. Furthermore, ROCK2 enhances the expression of adhesion molecules such as intercellular adhesion molecule-1 (ICAM-1) and vascular cell adhesion molecule-1 (VCAM-1) in endothelial cells [23], fostering a proinflammatory microenvironment that promotes leukocyte infiltration and perpetuates kidney injury.

ROCK2 also plays a central role in kidney fibrosis through several interconnected mechanisms. It amplifies TGF-β signaling, not via the canonical Smad pathway but through activation of mitogen-activated protein kinase (MAPK) cascades, particularly c-Jun N-terminal kinase (JNK) and extracellular signal-regulated kinase (ERK) [29]. These ROCK2-driven pathways enhance the expression of extracellular matrix proteins, including fibronectin and collagen IV, which accumulate in the glomerular mesangium and contribute to the characteristic histopathological changes of diabetic nephropathy. Notably, these findings have been corroborated by pharmacological studies: treatment with belumosudil, a selective ROCK2 inhibitor recently approved for the treatment of graft-versus-host disease (GVHD), has led to significant improvements in kidney histology and function in db/db mice, further supporting its therapeutic potential.

In podocytes, ROCK2 activation has particularly deleterious consequences, primarily through regulation of the Notch signaling pathway. Under diabetic conditions, ROCK2 promotes TGF-β-induced expression of Notch ligands, such as Jagged1, resulting in sustained Notch activation [17]. This ROCK2-Notch axis induces podocyte apoptosis by upregulating pro-apoptotic factors and downregulating survival pathways, contributing to podocyte depletion—a key driver of albuminuria and glomerular scarring in diabetic nephropathy. Loss of these terminally differentiated cells compromises the glomerular filtration barrier and initiates a cascade of pathological events that culminate in irreversible kidney damage.

Robust genetic evidence underscores the pathogenic role of ROCK2 in diabetic nephropathy. Podocyte-specific ROCK2 deletion in mouse models of both type 1 and type 2 diabetes preserves podocyte number, reduces glomerular fibrosis, and significantly attenuates albuminuria [6]. These protective effects are associated with elevated peroxisome proliferator-activated receptor α (PPARα) activation, diminished expression of apoptotic mediators, and normalization of energy metabolism within podocytes. The involvement of ROCK2 in kidney metabolism shows interesting parallels with ROCK1, likely due to their similar molecular structures. Both isoforms contain nearly identical kinase domains and regulatory elements, which may explain why they can both affect cellular energy regulation, even though they have distinct primary functions in disease processes. This structural resemblance helps us understand how ROCK2—best known for its roles in inflammation and scarring—can also participate in metabolic processes through mechanisms similar to ROCK1. Such dual functionality demonstrates how closely related protein structures can perform both shared and specialized tasks in kidney metabolism.

## Therapeutic implications of ROCK inhibition in CKD in people with diabetes

The growing understanding of ROCK signaling pathways in diabetic nephropathy has led to significant advances in developing targeted pharmacological interventions. Currently available ROCK inhibitors can be broadly categorized based on their isoform selectivity, each offering unique therapeutic potential for managing CKD caused by diabetes.

Pan-ROCK inhibitors represent the first generation of pharmacological agents targeting both ROCK1 and ROCK2 isoforms. Fasudil, the most clinically advanced drug in this class, has been approved in Japan since 1995 for preventing cerebral vasospasm following subarachnoid hemorrhage [30]. In preclinical models of diabetic nephropathy, fasudil has demonstrated remarkable kidney-protective effects, including a reduction in albuminuria, attenuation of glomerulosclerosis [12]. Its active metabolite, hydroxyfasudil, exhibits potent inhibitory activity against both ROCK1 and ROCK2, with comparable Ki values for the two isoforms. The research tool compound Y-27632, while not clinically approved, has been invaluable in elucidating ROCK biology in diabetic nephropathy. Ripasudil, currently approved as a topical agent for glaucoma treatment [31], represents another pan-ROCK inhibitor with potential systemic applications. While its systemic effects in CKD related to diabetes remain unclear, its favorable safety profile in ocular use suggests potential for broader therapeutic applications. The development of ROCK2-selective inhibitors has opened new avenues for targeted therapy in CKD among people with diabetes. Belumosudil is the most advanced ROCK2-selective inhibitor currently approved for clinical use, particularly in the treatment of GVHD [32].

These pharmacological agents exert their beneficial effects in diabetic nephropathy through multiple complementary mechanisms. At the glomerular level, ROCK inhibitors help restore filtration barrier integrity by inhibiting podocyte apoptosis [17]. They significantly reduce cell death by normalizing Notch signaling activity. The anti-inflammatory effects of ROCK inhibition on mesangial cells are mediated through suppression of NF-κB signaling and subsequent reduction in pro-inflammatory cytokine production [25]. Perhaps most importantly in diabetic nephropathy, these agents help restore metabolic homeostasis by improving AMPK signaling and mitochondrial function in kidney cells [33]. Their potent anti-fibrotic effects, achieved through inhibition of TGF-β signaling and extracellular matrix production, address one of the fundamental pathological processes driving diabetic nephropathy progression.

Emerging clinical evidence supports the therapeutic potential of ROCK inhibition in CKD among people with diabetes. A noteworthy retrospective study of patients with diabetes receiving fasudil for cerebrovascular indications demonstrated significant reductions in proteinuria, suggesting clinically relevant kidney protection [13]. This observation is particularly compelling as the reduction in proteinuria occurred independently of changes in blood pressure or glycemic control, highlighting the direct kidney benefits of ROCK inhibitor.

Statins, while primarily used for lipid management, may exert part of their pleiotropic kidney benefits through indirect ROCK inhibition. By blocking HMG-CoA reductase, statins reduce the production of isoprenoid intermediates necessary for Rho GTPase activation, thereby decreasing downstream ROCK activity. This mechanism may contribute to the observed kidney-protective effects of statins in patients with diabetes beyond their lipid-lowering actions [34]. These developments, along with ongoing clinical studies to assess the bioavailability of oral ROCK inhibitors [35], are paving the way for continuous approaches to the treatment of CKD in people with diabetes that directly address its molecular underpinnings.

As our understanding of ROCK isoform-specific biology in diabetic nephropathy deepens, the next generation of therapeutics will likely focus on achieving greater target specificity and tissue selectivity. The development of ROCK1-selective inhibitors remains an important unmet need that could provide valuable tools for targeting metabolic aspects of diabetic nephropathy. Additionally, innovative drug delivery systems designed to enhance kidney accumulation while minimizing systemic exposure may improve the therapeutic index of ROCK inhibitors for chronic use in CKD with diabetes. These advances, combined with a growing body of clinical evidence, position ROCK inhibition as a promising therapeutic strategy that may soon transition from bench to bedside in the management of CKD in people with diabetes.

The therapeutic potential of ROCK inhibition extends well beyond kidney diseases. Emerging clinical trials are investigating ROCK inhibitors for diverse pathological conditions, including amyotrophic lateral sclerosis (ALS), Alzheimer’s disease, and various ophthalmic disorders (Table 1), capitalizing on their pleiotropic mechanisms of action. While these studies may ultimately inform potential applications in nephrology, their current primary endpoints remain focused on non-diabetic indications. The convergence of these developments with accumulating clinical evidence establishes ROCK inhibition as a highly promising therapeutic paradigm that is rapidly transitioning from preclinical discovery to clinical implementation for managing CKD attributed to diabetes.

**Table 1:** Upcoming clinical trials of ROCK inhibitors

Disease	Interventions	Target	Phase	Identifier	Primary outcome
GVHD	Belumosudil	ROCK2	2	NCT07006506	Change in GVHD relapse-free survival at 1-year post-hematopoietic cell transplantation
Bronchiolitis obliterans	Belumosudil	ROCK2	2	NCT05922761	Overall response rate
Kidney transplant failure	Belumosudil	ROCK2	1	NCT05806749	Kidney allograft survival
ALS	Fasudil	ROCK1 and 2	2	NCT05218668	Incidence of adverse events
Ovarian cancer	Fasudil	ROCK1 and 2	2	NCT06890858	Overall objective tumor response rate
Alzheimer’s disease	Fasudil	ROCK1 and 2	2	NCT06362707	Cognition
Normal tension glaucoma	Netarsudil	ROCK1 and 2	4	NCT06449352	Mean diurnal intraocular pressure
Proliferative vitreoretinopathy	Netarsudil	ROCK1 and 2	2 and 3	NCT05660447	Single-surgery anatomic success rate
Fuchs endothelial dystrophy	Ripasudil	ROCK1 and 2	3	NCT05289661	Best spectacle-corrected visual acuity

*GVHD* graft-versus-host disease, *ALS* amyotrophic lateral sclerosis

## Expanding horizons: ROCK inhibition as a therapeutic strategy for focal segmental glomerulosclerosis

The therapeutic potential of ROCK inhibition has recently extended beyond diabetic nephropathy to include other proteinuric kidney disorders, with particularly promising applications in FSGS. This severe glomerular disease—characterized by podocyte injury and segmental scarring of the glomerular tuft—shares key pathogenic mechanisms with diabetic kidney damage, making it a compelling target for ROCK-directed therapies [7, 36].

In adriamycin-induced FSGS models, which closely replicate human disease, we have observed significant upregulation of ROCK2 specifically in podocytes. Genetic studies provide compelling evidence for the pathogenic role of podocyte ROCK2 in FSGS. Mice with podocyte-specific deletion of ROCK2 exhibit marked protection against adriamycin-induced kidney injury, maintaining normal glomerular architecture and function despite adriamycin exposure. These mice show significantly reduced albuminuria compared to wild-type controls, along with preserved podocyte numbers and minimal glomerular scarring. Histological analysis confirms intact foot processes and slit diaphragms in ROCK2-deficient podocytes, indicating that ROCK2 inhibition preserves the structural integrity of the glomerular filtration barrier under injurious conditions.

The molecular mechanisms underlying these protective effects involve a novel interplay between ROCK2 and cyclic nucleotide signaling pathways. Transcriptomic and proteomic profiling have revealed that ROCK2 inhibition upregulates regulator of G protein signaling 2 (RGS2), a key modulator of G protein-coupled receptor signaling. RGS2 expression is regulated via cyclic GMP-dependent protein kinase (PKG) signaling [37], uncovering an unexpected link between ROCK2 activity and cyclic nucleotide pathways in podocyte biology. Further mechanistic studies indicate that this ROCK2–RGS2 axis modulates cadherin-13 (CDH13) signaling, a pathway involved in cell adhesion and survival [38]. The net result is enhanced podocyte viability and resilience to injurious stimuli.

These preclinical findings have important therapeutic implications. The ROCK2-selective inhibitor belumosudil has demonstrated significant efficacy in FSGS animal models. Belumosudil treatment attenuates disease progression by reducing albuminuria, preserving podocyte number, and limiting glomerular fibrosis. These protective effects appear to arise from multiple mechanisms, including suppression of apoptotic pathways and inhibition of profibrotic signaling in glomerular cells. Importantly, the therapeutic benefits are evident even when treatment is initiated after disease onset, suggesting potential applicability in patients with established FSGS. Clinical studies are now required to determine whether these promising preclinical results translate into therapeutic benefit for patients with FSGS. Key considerations for clinical trial design include optimal dosing regimens, appropriate patient selection (potentially guided by ROCK2 activity biomarkers), and the use of meaningful outcome measures beyond albuminuria reduction. The development of non-invasive biomarkers to monitor ROCK2 activity and treatment response will be particularly valuable in facilitating clinical translation.

As this field advances, several critical questions remain. These include determining whether ROCK2 inhibition is most effective as monotherapy or in combination with existing treatments, assessing differential responses among FSGS subtypes, and identifying patient populations most likely to benefit from this targeted approach. Furthermore, the long-term effects of ROCK2 inhibition on kidney structure and function warrant careful evaluation in clinical settings.

The extension of ROCK-targeted therapy from diabetic nephropathy to FSGS marks an exciting development in the treatment of glomerular diseases. By targeting shared molecular drivers of podocyte injury and fibrosis, ROCK2 inhibition represents a promising precision medicine approach for a range of proteinuric disorders. As our understanding of ROCK isoform biology deepens, so too does the potential to develop more effective, mechanism-based therapies for FSGS and related glomerular pathologies.

## The emerging role of ROCK2 in kidney tubular electrolyte homeostasis

While considerable attention has focused on the glomerular actions of ROCK2 in kidney disease, emerging evidence highlights its equally important role in tubular electrolyte regulation, particularly in sodium handling. This tubular function represents a distinct yet complementary facet of ROCK2’s kidney activity, broadening our understanding of its contributions to overall kidney physiology in both health and disease.

Within the kidney tubules, ROCK2 has been identified as a novel regulator of mineralocorticoid receptor (MR) expression, acting through a signaling cascade involving signal transducer and activator of transcription 3 (STAT3) [39]. Studies using tubule-specific ROCK2 knockout mice have demonstrated that the genetic deletion of ROCK2 in tubular epithelial cells leads to markedly increased urinary sodium excretion, a finding that correlates with diminished MR expression in the kidney. Mechanistic investigations have revealed that ROCK2 maintains MR levels through STAT3-dependent transcriptional regulation. Specifically, ROCK2 promotes STAT3 expression, enabling STAT3 to bind the MR gene promoter and enhance its transcription. In the absence of ROCK2, this regulatory pathway is disrupted, resulting in reduced MR expression and decreased activity of the epithelial sodium channel (ENaC).

The physiological relevance of the ROCK2–STAT3–MR axis is particularly significant in pathological states characterized by sodium retention, such as diabetic nephropathy and salt-sensitive hypertension. In these conditions, elevated tubular ROCK2 activity may contribute to maladaptive sodium reabsorption via upregulated MR signaling. This mechanism may help explain the sodium retention frequently observed in patients with CKD [40], especially those with diabetes, where ROCK2 activation has been well-documented in the kidney. The sodium-retaining actions of ROCK2 could exacerbate volume overload and hypertension, thereby initiating a vicious cycle that accelerates kidney damage.

These findings have important therapeutic implications. Pharmacological inhibition of tubular ROCK2 presents a promising strategy for managing salt-sensitive hypertension in CKD by selectively modulating sodium handling in the distal nephron. Unlike traditional MR antagonists (e.g., spironolactone or eplerenone), which block MR activation by aldosterone, ROCK2 inhibitors would suppress MR expression itself. This could offer more complete attenuation of MR-driven sodium reabsorption. Such an approach could be particularly advantageous in CKD with diabetes, where ROCK2 inhibition might simultaneously alleviate glomerular inflammation and fibrosis while improving sodium homeostasis.

Nonetheless, several critical questions remain. The specific nephron segments in which ROCK2 exerts its predominant effects on sodium transport require further delineation, as does the relative contribution of this pathway in the context of other sodium-regulating mechanisms. In addition, potential interactions between ROCK2 and other transporters—such as the Na⁺/K⁺/2Cl⁻ cotransporter (NKCC2) or the sodium–hydrogen exchanger (NHE3)—warrant investigation. Clarifying whether ROCK2’s effects represent acute regulatory responses or longer-term adaptive changes is also essential.

The clinical translation of these insights will require careful consideration. Developing kidney-targeted ROCK2 inhibitors could optimize tubular efficacy while minimizing systemic effects, particularly those related to vascular tone. Combination therapies with existing antihypertensives may offer synergistic benefits and allow for dose reduction to limit adverse effects. Importantly, any kidney-protective effects achieved through ROCK2 inhibition must be weighed against potential disturbances in potassium handling, especially in patients with advanced kidney impairment.

The identification of ROCK2 as a regulator of tubular electrolyte homeostasis introduces a new dimension to its role in kidney pathophysiology. These findings suggest that the therapeutic benefits of ROCK2 inhibition may extend beyond its anti-inflammatory and antifibrotic effects to encompass improved volume regulation and blood pressure control. As research progresses, targeting ROCK2 may enable more precise interventions that address the intertwined processes of sodium retention, hypertension, and progressive kidney injury, particularly in the context of diabetic nephropathy, where ROCK2 activation contributes to multiple pathological pathways.

## Future perspectives and challenges

Although the therapeutic potential of ROCK inhibition in people with CKD and diabetes has been demonstrated in a preclinical study [13], several key challenges must be addressed to enable successful clinical translation. Moving forward, a careful and comprehensive approach is required—one that integrates scientific innovation, clinical insight, and therapeutic strategy.

A primary challenge lies in achieving enhanced isoform selectivity with pharmacological agents. Most current ROCK inhibitors lack sufficient discrimination between ROCK1 and ROCK2 [41], complicating efforts to delineate their contributions to disease progression. While genetic knockout models have provided valuable insights into the distinct roles of each isoform, the development of highly selective small-molecule inhibitors remains an unmet need. Recent advances in cryo-EM and AI-based drug design may enable precise targeting of isoform-specific structural features, such as ROCK’s unique C-terminal extension, potentially leading to more selective compounds. Such tools would not only advance mechanistic understanding but also enable the design of targeted therapies with improved efficacy and reduced off-target effects. Recent progress in structural biology and computational drug design offers promising avenues for developing next-generation isoform-specific inhibitors, particularly as our understanding of the subtle structural differences between ROCK1 and ROCK2 deepens.

Tissue-specific drug delivery represents another significant hurdle in the development of ROCK-targeted therapies for CKD in people with diabetes. Systemic ROCK inhibition—though effective in animal models—can lead to adverse effects, such as hypotension, due to ROCK’s established role in vascular smooth muscle tone regulation. Therefore, kidney-targeted delivery systems are urgently needed to enhance kidney exposure while minimizing systemic toxicity. Promising approaches include kidney-targeted nanoparticles encapsulating belumosudil using CD44-binding hyaluronic acid coatings [42], or antibody–drug conjugates targeting megalin in proximal tubules. Moreover, alternative administration routes, such as retrograde kidney venous infusion or intra-arterial delivery [43, 44], may offer enhanced organ specificity. These advances in drug delivery could significantly improve the therapeutic index of ROCK inhibitors and support their long-term use in patients with CKD.

The development of rational combination therapies is also essential. Given the multifactorial nature of CKD with diabetes, ROCK inhibition alone is unlikely to confer complete protection. Combining ROCK inhibitors with established therapies—such as sodium-glucose cotransporter-2 (SGLT2) inhibitors or renin-angiotensin system (RAS) blockers—may yield synergistic benefits by targeting complementary pathogenic mechanisms. For instance, co-administering ROCK and SGLT2 inhibitors could simultaneously address hemodynamic disturbances and proinflammatory signaling. Preclinical studies are needed to evaluate dosing strategies, potential interactions, and sequencing protocols to maximize therapeutic outcomes. Furthermore, the development of fixed-dose combinations or co-formulations may improve adherence and optimize treatment effectiveness in clinical practice.

A critical gap in current research is the lack of robust, accessible biomarkers to monitor ROCK activity and therapeutic response. Reliable biomarkers would facilitate patient stratification, guide dose selection, and enable real-time treatment monitoring. Potential candidates include urinary or plasma indicators of ROCK-mediated phosphorylation events, microRNA signatures linked to ROCK activation [45], or non-invasive imaging modalities capable of quantifying kidney fibrosis. Integrating multi-omics data—such as proteomics, transcriptomics, and metabolomics—with clinical parameters may aid in identifying predictive and pharmacodynamic biomarkers to support personalized therapy in patients with CKD and diabetes.

Finally, large-scale, rigorously designed clinical trials are essential to establish the safety and efficacy of ROCK inhibitors in human populations. While preliminary studies with fasudil have shown reductions in proteinuria, these findings must be confirmed in randomized controlled trials with kidney endpoints, such as estimated glomerular filtration rate (eGFR) slope or kidney survival. Trial design should carefully consider inclusion criteria (e.g., disease stage or biomarker profiles), optimal dosing, and relevant outcome measures beyond proteinuria. Long-term safety monitoring is particularly important to assess potential effects on blood pressure, hepatic function. International collaboration may be necessary to achieve the sample sizes and follow-up durations required for definitive evaluation.

Overcoming these challenges will demand sustained, multidisciplinary collaboration among basic researchers, clinicians, pharmacologists, and industry stakeholders. The potential reward is substantial: the development of a novel class of kidney-protective therapies that could meaningfully alter the course of diabetic nephropathy and potentially other forms of CKD. As the field progresses, ROCK-targeted therapy may soon become a clinical reality, transforming the management of this serious complication of diabetes.

## Conclusion

The small GTPase–ROCK signaling axis has emerged as a central regulator in the pathogenesis of diabetic nephropathy, integrating metabolic, inflammatory, and fibrotic pathways. Isoform-specific functions of ROCK1 and ROCK2 contribute to complementary yet distinct pathogenic mechanisms across glomerular and tubular compartments. ROCK1 primarily governs metabolic disturbances and mitochondrial dysfunction, whereas ROCK2 predominantly mediates proinflammatory and profibrotic responses. These insights, along with recent discoveries regarding ROCK2’s roles in FSGS and tubular electrolyte regulation, underscore the therapeutic promise of isoform-selective ROCK inhibition. As pharmacological strategies continue to advance, targeting the ROCK pathway may offer novel opportunities to address the rising global burden of CKD in people with diabetes. Future research should prioritize the clinical translation of these molecular findings, with the ultimate aim of improving outcomes for patients with CKD with diabetes and other related kidney disorders.
